# Experimental Study of Smart Steel Cables with Tubular Spot-Welded Grating Sensors

**DOI:** 10.3390/s25072148

**Published:** 2025-03-28

**Authors:** Nianchun Deng, Zhongqing Han, Zhiqian Chen, Zhaotao Chen

**Affiliations:** 1 School of Civil Engineering and Architecture, Guangxi University, Nanning 530004, China; dengnch@gxu.edu.cn (N.D.); 2310302030@st.gxu.edu.cn (Z.C.); 2 Guangxi Road and Bridge Engineering Group Co., Ltd., Nanning 530200, China; cztlq2025@163.com

**Keywords:** tubular spot-welded grating sensors, smart steel strand cable, cable force values, strain performance, temperature compensation

## Abstract

In this study, a tubular spot-welded grating sensor composed of a stainless-steel tube fixed to a substrate surface by welding is developed, and the tube is filled with high-performance epoxy resin components after the grating sensor is passed through it. A smart steel strand cable is created by spot welding steel strands using portable spot-welding equipment. This method generates a small current during spot welding, with a voltage of only 3 V to 5 V, and does not damage the internal structure of the steel strand. An equation related to the temperature, tension force, and wavelength fluctuation is presented in this article. A method with a transverse temperature coordinate and a longitudinal wavelength coordinate is used. A formula for the standard temperature calibration of the force values and a procedure for temperature adjustment of the force values are presented. The correlation coefficient between the stress on the steel strand and the wavelength of the tubular spot-welded grating sensor is as high as 0.999 according to static tensile testing, demonstrating good repeatability. The temperature adjustment coefficient for varying temperatures is 0.0264 nm/°C, and the test error is essentially limited to 3.0% F.S. When subjected to a 120 h relaxation test, the steel strand with the tubular spot-welded grating sensor exhibits a relaxation rate of 4.44%. The force value obtained after the relaxation test is 1.2% off from the standard load. A tubular spot-welded grating sensor is welded onto a steel strand within a cable sealing cylinder to create an extruded anchor epoxy-coated steel strand cable. The measured cable force is compared with the standard load. The maximum error is 0.5% F.S. The discrepancy between the measured cable force and the acceleration sensor value is 1.5% in one instance involving an arch bridge employing six smart suspension cables to detect cable forces onsite. The findings provide theoretical and engineering references for smart cables and demonstrate the high accuracy, dependability, and fixation performance of the tubular spot-welded grating sensor smart cable.

## 1. Introduction

The most important component of cable-supported bridges, such as cable-stayed suspension bridges and cable-stayed arch bridges, is the bridge cable, of which the durability and dependability are closely related to the safe operation, upkeep, and service life of the bridge. However, in-service bridge cables are deteriorating daily and are damaged to varying degrees, meaning that their service life will only be between 35% and 60% of their design life [1,2]. The breaking of bridge cables frequently results in serious accidents involving the collapse of bridges. A reasonable and reliable test method that can not only resolve the issue of cable force construction monitoring for cable-supported bridges but also monitor the health and safe operation status of bridges in the future will have strong application value [3,4]. A sensor is a crucial instrument for monitoring cable construction and health. Currently, bridge cable static, dynamic, and temperature measurements, as well as the detection of bridge cable damage, can all be accomplished with high precision using fiber Bragg grating (FBG) sensors [5,6]. Nevertheless, because of the makeup of the optical fiber, its shear resistance is low and its materials are delicate. Research on smart cables has focused on how to safely install fiber-containing grating sensors on steel wires, steel cables, parallel steel wire ties, stranded wire ties, and other structures to create self-sensing smart cables [7,8].

The two primary types of optical fiber smart cables currently being researched are fiber grating smart parallel wire cables and fiber grating smart steel strand wire cables. Some of the fiber grating arrangement techniques are as follows: (1) To create fiber-reinforced polymer (FRP)-optical FBG (OFBG) bars, fiber-containing OFBGs are inserted in the center of the bars along their length. The FRP-OFBG bar and steel wire are combined to form a parallel steel wire cable. Alternatively, steel wires in steel strands can be replaced with fiber optic smart structures to create fiber optic smart strands, which can then be integrated into parallel steel strand ties by combining steel strands and fiber optic smart strands. A zip wire is used to monitor the cable force and evaluate damage by anchoring it in an anchorage [9,10,11,12]. The drawback of this arrangement is the difficulty of the FRP-OFBG fiber bonding procedure. (2) To develop fiber grating sensors, grooves are carved into steel wires. Steel strands are interwoven in parallel using fiber optic grating steel wires with grooves. A fiber optic smart strand is created by substituting the steel wires in steel strands with grooved fiber grating steel wires. A steel strand and a fiber optic smart strand are then connected to create a parallel steel strand cable [13,14,15,16]. The drawback of this arrangement method is that the fiber grating is placed in the groove of the wire, which increases the likelihood that it will fall off as the glue ages, and it will be damaged more easily because it is exposed. (3) Conventional bonding and the unique hoop (or clamp) connection are two techniques used to connect a wire in a cable to a particular fiber grating strain sensor [17,18,19]. The drawback of the conventional glue connection is that as the glue ages, the connection will become loose. The steel wire is easily loosened, so securing the steel wire and the grating sensor when it is connected to the steel wire or steel strand using a hoop (or clamp) connection is challenging. Moreover, the way the fiber grating is arranged above does not account for temperature variations.

In addition to studying the measurability and reliability of fiber gratings, appropriate embedding techniques must be developed to fully exploit the potential of built-in fiber gratings. The specifications for “Hot-dip galvanized steel wires for bridge cables, Standardization Admin” [20], “prestressed concrete with steel wire” (GB/T5223) [21], and “prestressed concrete with steel strand” (GB/T5224) [22] state that broken wires can be easily formed at the welding sites under tension because the welding strength is significantly lower than the strength of the steel itself. The idea presented in this study is to fuse steel wires and steel strands to a tubular spot-welded grating sensor. The idea is to modify the welding machine to use electric welding, which is based on the double-sided double-point overcurrent welding principle. Under small spot welding current and voltage (only 3–5 V), when the welding current flows from one electrode to the other electrode, the two contact resistance points form an instant hot melt contact, and welding is accomplished. The welding current instantly passes from one electrode to the other electrode, forming a loop and passing between the two workpieces without causing any damage to the internal structure of the welded workpiece [23]. To create a smart cable, a tubular spot-welded grating sensor is built in this study and spot-welded to a steel strand. Through temperature testing, a temperature compensation technique study, strand relaxation influence testing, testing of spot welding between sensors and strands, and testing of the spot-welded grating sensor and strand cable performance, the performance and engineering application of smart cables are investigated.

## 2. Fiber Bragg Grating Basic Principles and Tubular Spot-Welded Grating Sensor

A typical type of fiber grating is the FBG. The refractive index of the optical fiber core is periodically modulated axially to generate a diffraction grating. This passive filter has a small reflection band. Incident light of a certain wavelength can be partially or fully reflected by the fiber grating. Variations in the Bragg wavelength are caused by changes in the temperature and stress (strain). The following describes the relationship between temperature, strain, and wavelength drift [24].

(1) $$\frac{\Delta \lambda }{\lambda }=(1-P)\varepsilon +\left(\xi +\alpha \right)\Delta T$$

where *ε* represents the axial strain, ΔT represents the temperature difference, *P* represents the effective photo-elastic coefficient, *ξ* represents the thermal optical coefficient, and *α* represents the thermal expansion coefficient.

In the formula, the total wavelength variation is composed of the change caused by force and that caused by temperature. The center wavelength change due to force is represented by (1−P)ε, and the strain—induced change is $\left(\xi +\alpha \right)\Delta T$. Here, $\xi =\frac{1}{n}\frac{dn}{\mathrm{dT}}$, $\alpha =\frac{1}{\Lambda }\frac{d\Lambda }{\mathrm{dT}}$, with *n* being the refractive index and Λ the fiber grating period.

The fiber grating sensor is lightweight, has a small volume, is flexible, and has little influence on the measured medium. The fiber grating sensor has little influence on the stress and strain of the component. When the fiber grating is in a uniform strain field, the stress calculation formula for the component at a certain position is as follows:

(2) $$\sigma =\frac{E}{1-P}\left[\frac{\Delta \lambda }{\lambda }-\left(\xi +\alpha \right)\Delta T\right]$$

The relationship among the component force, wavelength increment, and temperature variation is:

(3) $$F=k_{f}\Delta \lambda +\alpha_{T}\Delta T$$

where $k_{f}$ is the wavelength change sensitivity coefficient, Δλ is the wavelength increment, $\alpha_{T}$ is the temperature sensitivity coefficient, and ΔT is the temperature variation.

**Figure 1:**
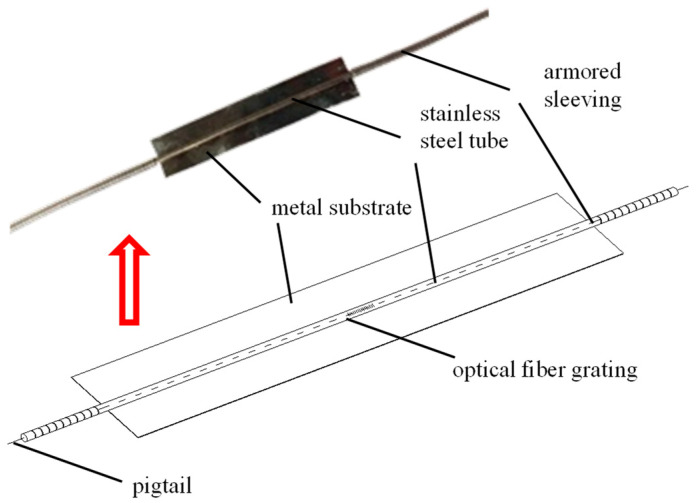
Tubular spot-welded grating sensor.

**Figure 2:**
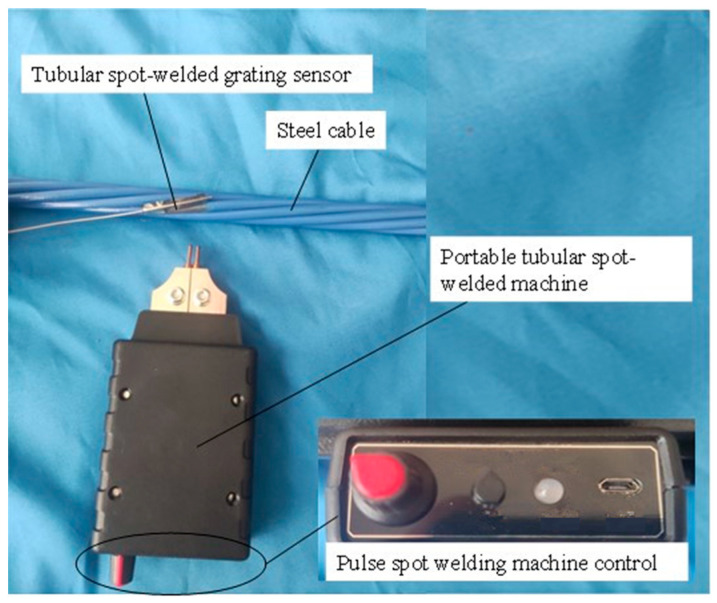
Portable tubular spot-welded machine and welding process.

The components of the tubular spot-welded grating sensor include a metal substrate, a fiber optic grating, a protective armored casing, and a stainless-steel tube [25]. The stainless-steel tube is 40 mm long, 0.8 mm in diameter on the outside, and 0.1 mm thick. The metal substrate is 0.05 mm thick, 7 mm wide, and 35 mm long. The coating layer of the 125 µm diameter fiber is removed to create the fiber grating. The grating measures 10 mm in length and 10 µm in core diameter. The following is the precise procedure for building the tubular spot-welded grating sensor. The grating sensor is passed through the stainless-steel tube after the stainless-steel tube has been spot-welded onto the broader side of the stainless-steel substrate. To fix the sensor, two high-performance epoxy resin components are injected into the tube. Afterward, the stainless-steel pipe and the armored casing are sealed and protected. The fiber tail must be extended, welded, and equipped with an optical fiber connection. The particular structure is displayed in Figure 1. The welding machine is a portable pulse welding machine with a built-in micro lithium battery and a working voltage ranging from 3 to 5 V. Figure 2 shows the structural drawing and welding procedure.

The tubular spot-welded grating sensor is packaged using the following procedure: (1) After being cleaned, the stainless-steel tube and substrate are placed on the spot-welding equipment. Spot welding produces a relatively low electric current between 3 and 5 V and does not harm the interior structure. (2) Then, the grating sensor is passed through the stainless-steel tube, and the tube is filled with high-performance epoxy glue. (3) After the temperature is set to 80 °C, the device is placed in an incubator for four hours to dry. It is then heated to a certain temperature. The two main factors considered during curing are the drying temperature and duration.

## 3. Shear Test for a Metal Substrate with Spot-Welded Steel Wires

**Figure 3:**
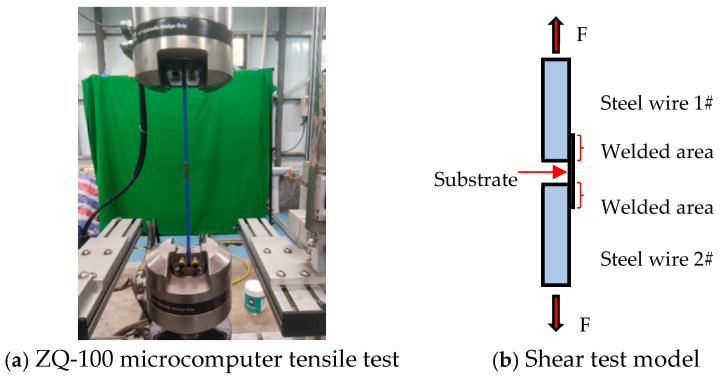
Shear test for a stainless-steel substrate with spot-welded steel wires.

Two epoxy-coated steel wires (7 mm in diameter and 400 mm in length) and one thin stainless-steel sheet (35 mm × 7 mm × 0.05 mm) were spot-welded together with a portable spot welder for shear tests on a ZQ-100 microcomputer tensile testing machine ((Dongguan Zhicai Precision Instrument Co., Ltd., Dongguan, China) shown in Figure 3a), and the model is shown in Figure 3b. This was done to investigate the shear resistance of spot-welded steel wires with epoxy coatings on stainless-steel substrates. Three shear tests were conducted in total. As the thin steel sheet was being de-welded, the wire tension was noted, and the shear stress at the weld junction was calculated. Table 1 displays the test findings. The average shear stress at the weld joints was determined by the tests to be 37.2 MPa.

## 4. Experimental Study on the Mechanical Performance of Smart Cables with Tubular Spot-Welded Grating Sensors

### 4.1. Force Sensing Performance of the Smart Steel Strand Wire

**Figure 4:**
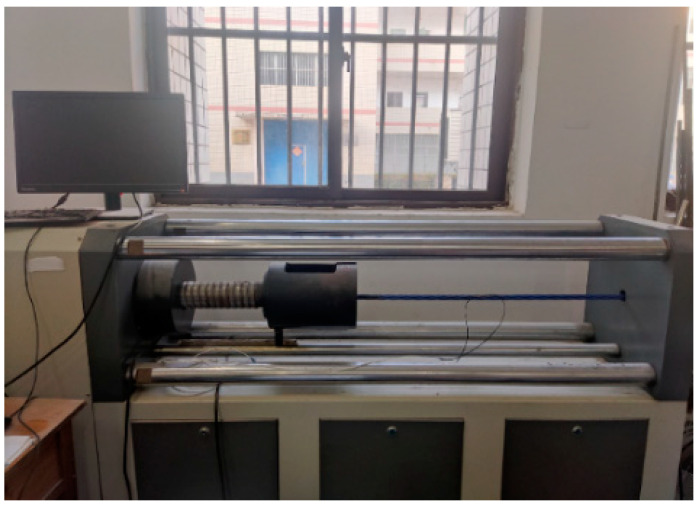
Testing the efficacy of tubular spot-welded grating sensors for strain sensing.

**Figure 5:**
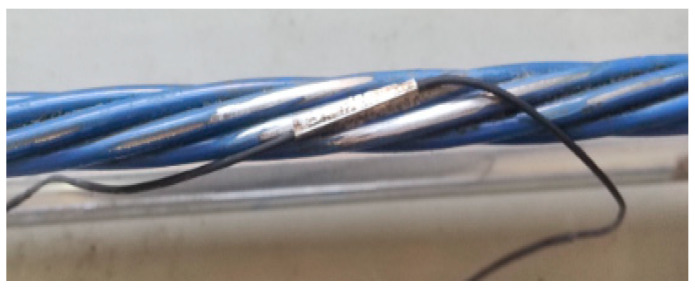
Schematic diagram of the strain performance test sample of a tubular spot-welded grating sensor.

The test was implemented on a 7-wire strand with a diameter of 15.2 mm, and the tensile strength, elastic modulus, and nominal breaking force were 1860 MPa, 195 GPa, and 260.4 kN, respectively. A portion of the cable body was polished in the steel wire direction. The tubular spot-welded grating sensors were welded and installed following alcohol-based cleaning and swabbing of the cable body. The portable spot welder was set to operate at an appropriate current, and two electrodes were brought into contact with the grating sensor edge [26,27]. By using the thermal effect of the resistance at the point of contact, thermal fusion welding formed a junction that solidified upon cooling. Generally, the weld joint spacing was kept at 0.8 mm, and the weld joints were crisscrossed in position. The steps involved in the smart cable spot welding sequence were as follows: (1) weld upward along the length of the spot-welded grating sensor at the center; (2) weld upward along the other side of the sensor at the midpoint; (3) weld downward along the sensor at the weld starting point in the first step; and (4) weld downward along the sensor at the starting point in the second step until the end. A smart cable test was conducted on a static tensioning platform [28]. As illustrated in Figure 4 and Figure 5, the test setup consisted of a 300 kN standard pressure ring transducer and accompanying instruments, a spot-welded grating sensor smart strand, a strand anchorage, a GM8050E (Guilin Guangming Technology Industrial Co., Ltd, Guilin, China) grating demodulator, etc.

The loading steps were as follows. Prior to being unloaded to 0.04 Pb, the cable was first pretensioned to 70% of the nominal breaking force Pb. To remove the geometric nonlinearity resulting from the space between the smart cable, tensioned workpiece, and tensioning tool, this cycle was repeated three times. Next, tensioning was begun at 0.04 Pb. Every load level had a duration of five minutes, the loading speed was 70 MPa/min, and each tensile load level was 0.115 Pb. Next, the data were recorded and read by the grating demodulator. A conventional pressure ring sensor regulated the test load.

**Figure 6:**
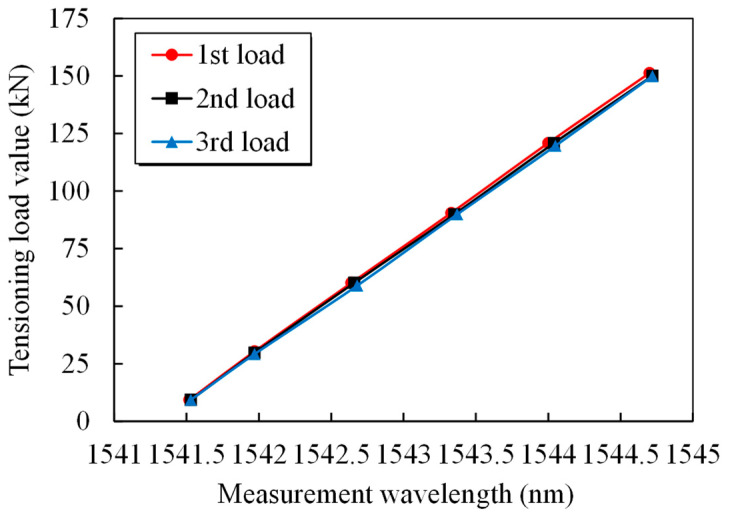
Relationship between the grating wavelength and steel strand loading.

**Figure 7:**
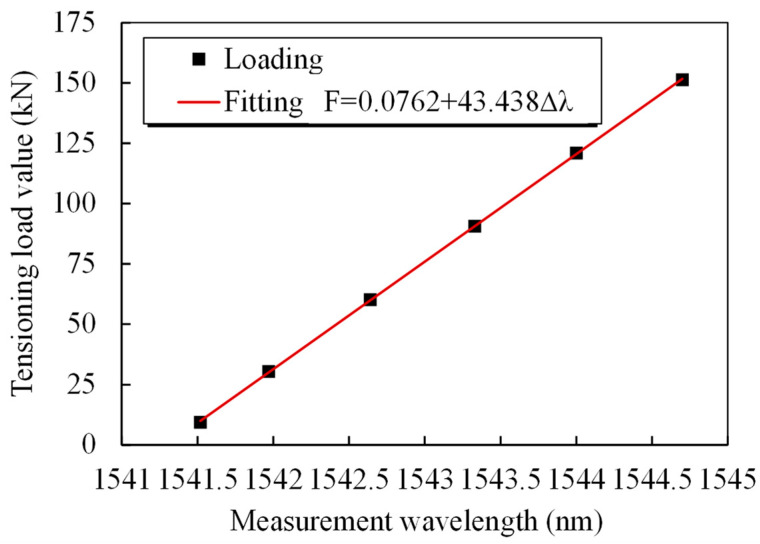
Data-fitting curves for the grating wavelength and steel strand test.

According to theoretical Formula (2), there is a linear relationship between the smart steel strand force and the grating wavelength. Figure 6 and Figure 7 illustrate how the grating wavelength and steel strand loading data under three loading cycles linearly match the fitting formula f=0.076+43.438Δλ. Figure 6 and Figure 7 clearly show that the tubular spot-welded grating sensor is undamaged, the steel strand is not ripped out, and the sensing function is stable. The smart cable could detect over 70% of the strand’s nominal breaking force. A fixed linear relationship exists between the applied load and the grating wavelength. The test results demonstrate the steady and dependable adhesion of the tubular spot-welded grating under cyclic loading, as well as the good repeatability of the tubular spot-welded grating sensor in the smart cable. The discrepancy between the theoretical force and the measured standard force is maintained within 2.3%, demonstrating that smart steel cables equipped with tubular spot welded grating sensors possess reliable and high-precision monitoring capabilities, as shown in Table 2.

### 4.2. Temperature Test for Steel Cables and Compensation Method

**Figure 8:**
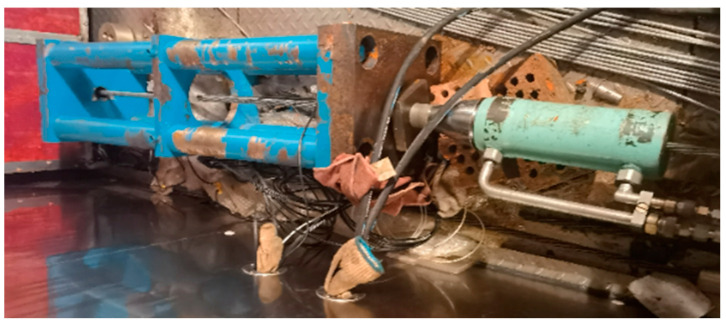
Temperature test setup.

The epoxy-coated steel strand was subjected to several repeated-loading tests utilizing a test rig in a constant-temperature test chamber to examine the temperature characteristics of the tubular spot-welded grating sensor [29]. Figure 8 depicts the temperature laboratory testing setup. The temperature was changed every ten degrees between −20 and 50 degrees. The loading regime was consistent with that of cyclically tensioned smart cables in Section 4.1.

**Figure 9:**
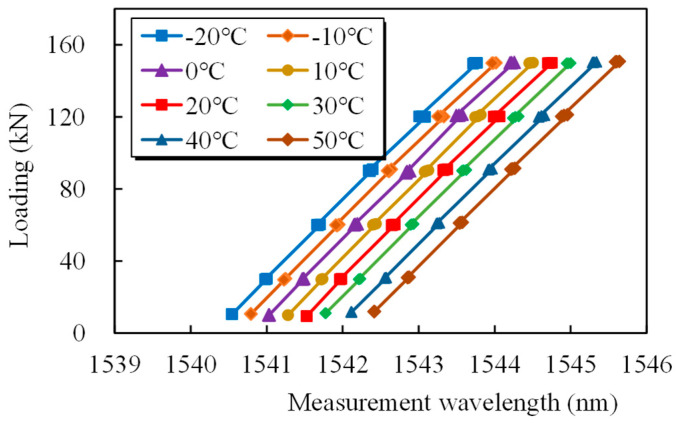
Relationship between the grating wavelength and load at different temperatures.

**Figure 10:**
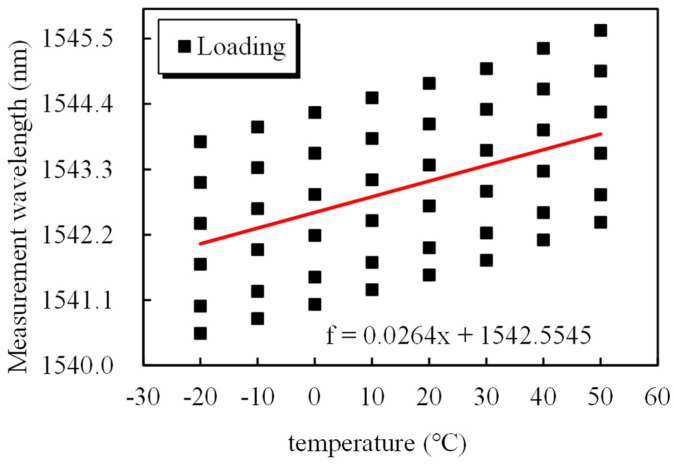
Fitting relationship between the grating wavelength and temperature.

Figure 9 illustrates the excellent linear relationship between the measured tensile load and the grating center wavelength over a wide temperature range, as well as the excellent reproducibility of the test findings. The wavelength–load relationship curves have roughly equal slopes at all temperatures, indicating that temperature changes only affect the curve intercept, or the grating wavelength in the absence of a load, and have no effect on the load sensitivity. The results of the temperature sensitivity test of the smart steel strand wire cable are shown in Figure 10. As shown in Figure 10, the load fluctuates with temperature, and the wavelength can sustain almost the same increment, suggesting that temperature has little bearing on the tubular spot-welded grating’s load sensitivity.

Taking 20 °C as the standard temperature, the relationship between the load and wavelength can be obtained by linearly fitting the 20 °C data in Table 3: f=43.456ΔT+0.298. We assume that T_0_ is the standard temperature, which is 20 °C. The wavelength at the zero point, or *λ*_0_, is 1541.304 nm. Under different temperatures and tensile loads, a method involving the set force, a transverse temperature coordinate, and a longitudinal strain coordinate is proposed, in which linear fitting is performed via the least-squares method. A right-angle coordinate system in which the wavelength is the vertical coordinate and the temperature is the horizontal coordinate is created. This coordinate system is used to label the data of each point in Figure 10, and the least-squares method is then applied to fit the data and produce the linear fitting curve: f=0.0264ΔT+1542.555. The slope of this straight line is the temperature compensation coefficient α_T_, which is equal to 0.0264 nm/°C. The temperature compensation for the wavelength is $\Delta \lambda =\left(\lambda -\lambda_{0}\right)+0.0264\left(T-T_{0}\right)$. Then, the temperature compensation relationship for the force of the smart steel strand cable is as Formula (4). Table 4 presents a comparison of the calculated results and the standard results. Overall, the applied force increases with temperature, as evidenced by the data.

(4) $$f=43.4557\left(\Delta \lambda -0.0264\left(T-T_{0}\right)\right)+0.2979$$

### 4.3. Tests on the Stress Relaxation of the Steel Strands

For the relaxation test, a typical steel strand twisted from seven steel wires with a nominal diameter of 15.2 mm and a tensile strength of 1860 MPa was utilized. References GB/T5224 [22] and GB/T21839-2019 [23] provide the test technique and judgment basis for the stress relaxation test of steel strands, respectively. The test procedure involves applying an initial force, typically 70% of the maximum force of the sample, to the specimen within the typical temperature range of 20 °C ± 2 °C. Under the action of this force, the sample undergoes elastic deformation. The internal stress decreases as a result of elastic deformation turning into inelastic deformation as the force duration increases, and the sample stays at a specific length. The apparatus automatically tracks the sample force variations. Reference GB/T5224 [22] states that the test data at 120 h can be used to calculate the relaxation rate at 1000 h.

**Figure 11:**
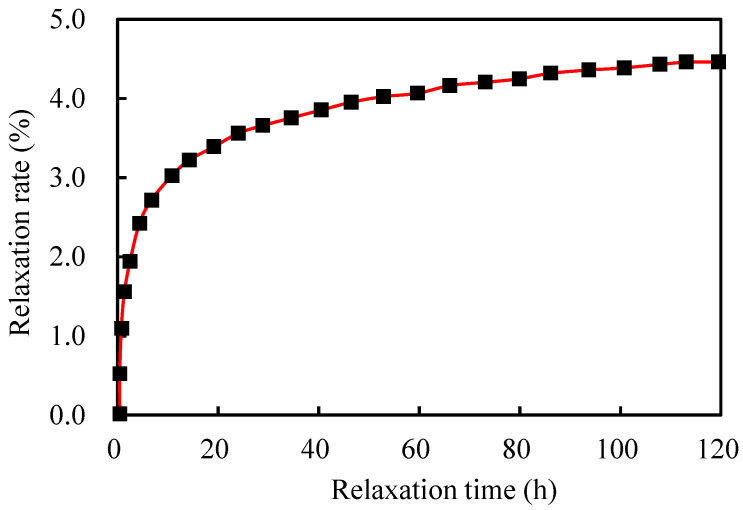
Relationship between the relaxation rate and time.

**Figure 12:**
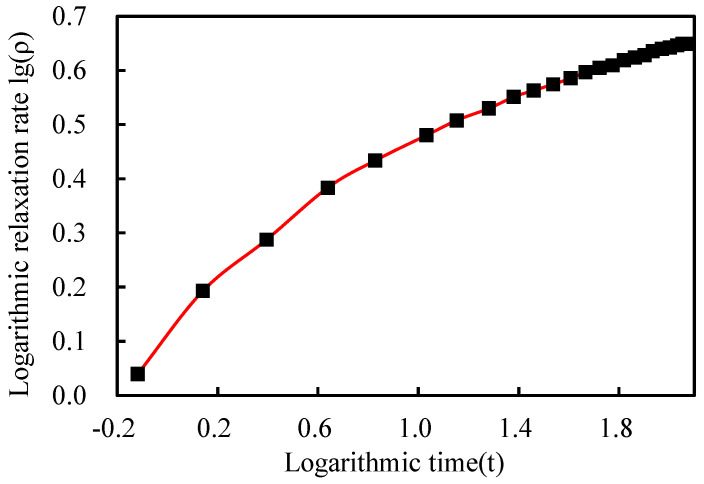
Relationship between the logarithm of time and the logarithm of the relaxation rate.

During the test, apparent temperature changes will cause a significant variation in the strand relaxation rate as well as a change in the starting force maximum value in strand loading under constant strain conditions [30]. As a result, during the test procedure, the temperature needs to be tightly maintained at 20 °C. Once the initial force reaches 80%, the remaining 80–100% of the force is constantly loaded within 2 min. The first 20% of the force is loaded as needed. The remaining 20–80% of the force is continuously loaded within 6 min. When the initial force is reached, the force remains unchanged for 5 min. Changes in the time and force need to be recorded during the loading process. The relaxation rate at 1000 h is extrapolated using the relaxation test value at 120 h, and reference GB/T21839-2019 [23] gives the following extrapolation formula:

(5) lgρ=mlgt+n

where ρ is the relaxation rate/%; t is the time/h; and m and n are coefficients. The data were recorded at 1 min, 2 min, 3 min, 4 min, 5 min, 8 min, 10 min, 15 min, 30 min, 45 min, 1 h, 2 h, 4 h, 8 h, 16 h, 24 h, 48 h, 72 h, 96 h, and 120 h. The obtained relaxation test data, as shown in Table 5, indicate that the strand displacement remains unchanged during the test, the test force gradually decreases, and the relaxation rate gradually levels off. After 120 h, the remaining test force is 172.1 kN, and the test relaxation rate is 4.44%. As shown in Figure 11 and Figure 12, the relationship between time and relaxation rate is determined using the logarithm of time as the horizontal axis and the logarithm of the relaxation rate as the vertical axis. Equation (4) illustrates how the least-squares approach is used to generate the equation for the relaxation rate as a function of time following linear regression. Equation (6) yields a relaxation rate of approximately 6.32% at 1000 h.

(6) lgρ=0.156⋅lgt+0.333

Table 6 displays the results of the relaxation process tests conducted on steel strands with tubular spot-welded gratings before and after loading for one minute and one hundred hours. The relaxation rate obtained from the derived formula is compared with the actually measured relaxation rate, as shown in Table 6. After being subjected to long-term stress, the estimated and actually measured relaxation rates of 0.044 show that the tubular spot-welded grating sensors can properly monitor the internal forces and deformations of the cable.

The multiple cyclic tensioning data of the smart cable before the relaxation test were fitted to obtain the fitting formula F=45.505Δλ, and the zero point was 1537.111. The standard load is 172.1 kN and the force determined after relaxation is 170.0 kN. Based on the 2.1 kN difference and 1.2% inaccuracy, the tubular spot-welded grating sensor can still guarantee a strong welding bond and steady operation even under heavy loads over an extended period of time.

### 4.4. Smart Steel Strand Cable Performance Test

**Figure 13:**
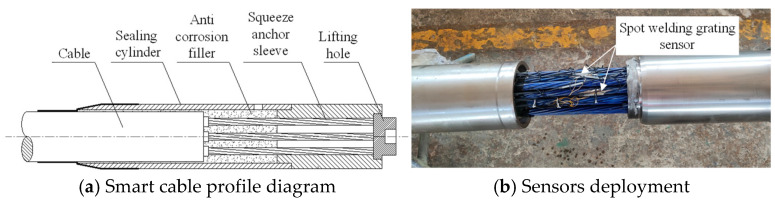
Smart steel strand cable.

**Figure 14:**
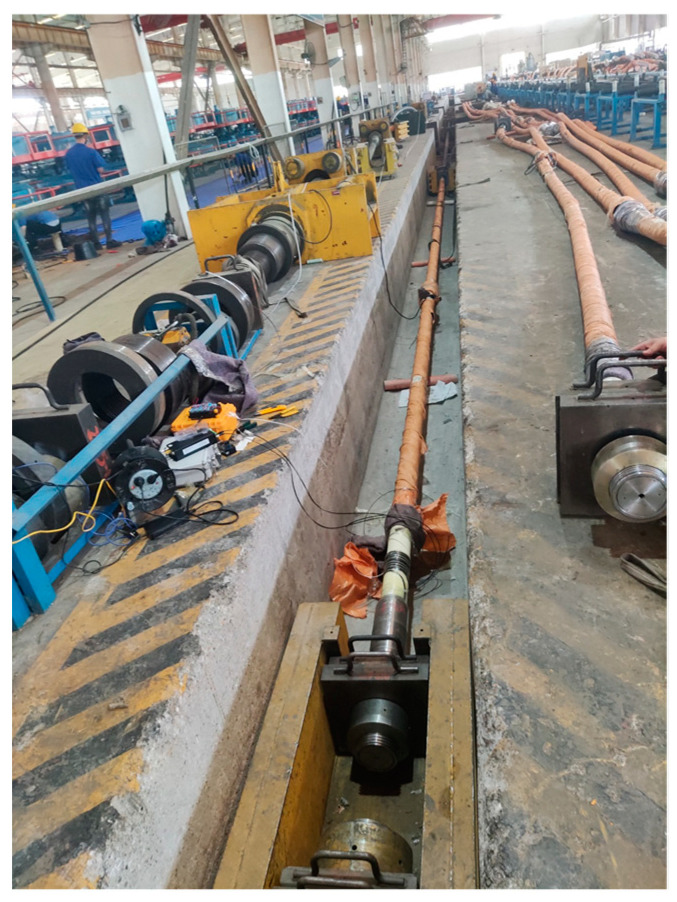
Static test of the smart steel strand cable.

To explore the smart strand strain synergism and performance, an extruded steel strand smart cable was fabricated and subjected to a static load test. A 34.3 m long steel fiber smart cable was created, with an epoxy-coated 25×15.2 mm steel strand serving as the cable body. The outside diameter *σ*_b_ of the cable body is 145 mm, with a steel strand extruded polyethylene (PE) sheath. The nominal breaking force of the strand is 6510 kN, its design force is 3130 kN, and its tensile strength P_b_ is 1860 MPa. A stranding machine spirally twisted 25 pieces of high-strength epoxy-coated seven-strand steel wires at a specific twisting distance to create the cable specimen. A hot-extruded high-density PE (HDPE) sheath and high-strength polyester tape were used to wrap it. The body of the cable was cemented to the anchorage by extrusion to form the cable assembly. An extruded anchoring sleeve, a corrosion-resistant material, a sealing device, a nut, and other components compose the cable anchorage assembly [31]. As shown in Figure 13b, the tubular spot-welded grating sensor is spot-welded to the steel strand inside the sealing device. The test specimens for the steel strand smart cable are outfitted with tubular spot-welded grating sensors. Each smart cable anchorage has one spot-welded temperature sensor and two attached tubular spot-welded grating sensors. The sensor leads are routed from the steel sleeve waterproof cover. The performance test of the cable was carried out in the tensioning test tank of the cable production base of Liuzhou OVM Machinery Co., (Liuzhou, China) as shown in Figure 14. The standard force transducer was the CL-YB-12MN anchor force transducer model, which had a maximum range of 12,000 kN and an uncertainty of 0.02.

**Figure 15:**
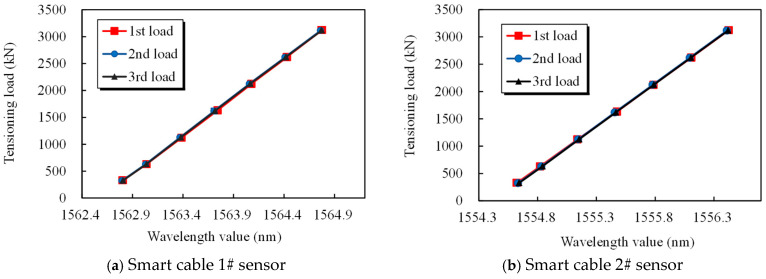
Relationship curve between the loaded strain and wavelength for the smart cable.

The loading steps were as follows. First, pretensioning was performed three times in the tie cable. After the smart tie was loaded to 0.5 times its normal breaking force (Pb), it was unloaded to zero. Next, loading began at zero, and 0.08 Pb was loaded at a time in each step. The load was held constant for five minutes after applying a loading rate of no more than 100 MPa/min, gradually increased to 0.5 Pb, maintained at this level for ten minutes, and then unloaded to zero. Afterward, formal loading was begun using the following loading procedures. Loading from 0 to 0.6 Pb began. The load was 0.08 Pb per stage, and the strain of the spot-welded grating sensor was recorded after the load was held for 5 min per stage. The anchor force transducer between the jack and the tensioning platform measured the cable force in the smart cable static tensile load calibration test. Figure 15 displays the observed wavelength vs. load relationship graph and illustrates how the data points roughly follow a straight line. The standard load in the cable force measurement and the computed load are compared in Table 7 to confirm the correctness and dependability of the smart cable. Table 7 shows that there is a maximum discrepancy of 0.5% F.S. between the estimated cable force of the steel strand smart cable and the observed cable force, which indicates that the tubular spot-welded grating sensor smart cable has a high degree of accuracy and reliability.

### 4.5. Engineering Applications

**Figure 16:**
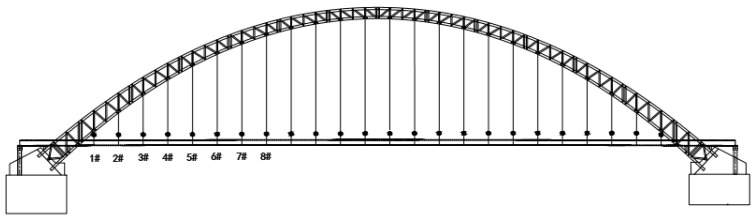
Mid-through concrete-filled steel tube arch bridge.

**Figure 17:**
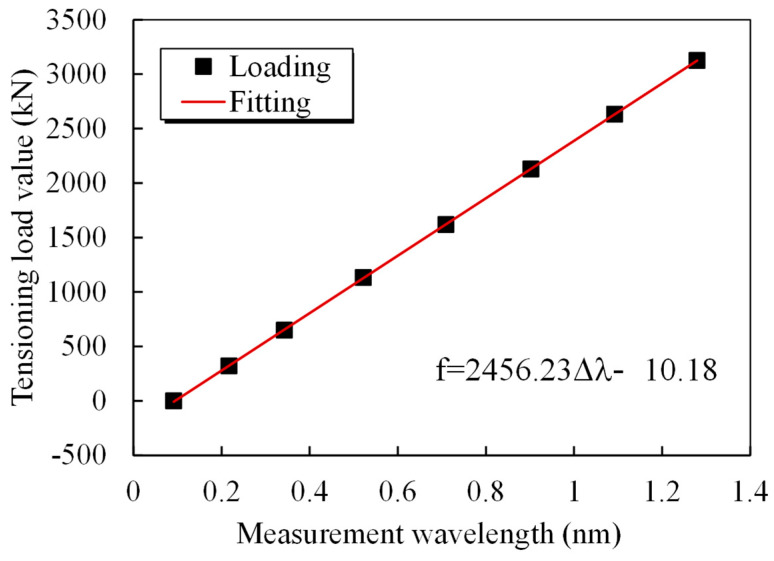
Calibration fitting curve of the cable force of s#1.

**Figure 18:**
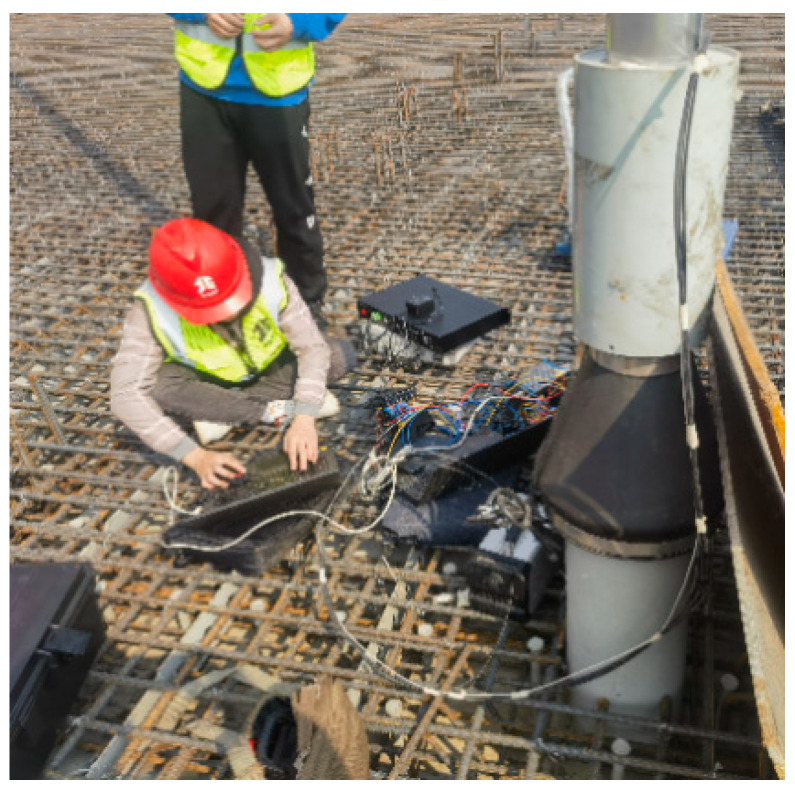
Measurement site of the cable force of the arch bridge.

A mid-through, 354 m main-span steel tube arch bridge made of concrete is considered, as shown in Figure 16. With a deck width of 18.9 m, the mainline bridge is a two-lane, two-way secondary traffic bridge. There are 28 parts in the steel pipe arch rib, and there are 12 sections in the cross-link. The bridge uses an extensive extruded strand cable system in addition to a steel-tube-concrete truss structure. Steel lattice girders support the primary girder structure of the integrated steel and concrete deck slab. Concrete was used to cast the arch footing. Upstream 1#, upstream 2#, upstream 3#, downstream 1#, downstream 2#, and downstream 3# utilize smart tensioning cables with tubular spot-welded grating sensors. The bridge cables were tested under tension when they left the factory. An anchor force sensor was adopted as the standard sensor. The relationship between the boom strain and the anchor force was calibrated, and the force calibration formula and the field measurements of the cable forces are shown in Table 8. Figure 17 shows the calibration fitting curve of the s#1 cable force. The calibration method is based on the fitting formula of the average wavelength and force of the two spot-welded grating sensors on each cable. The measurement site of the cable force on the suspension cables of the arch bridge is shown in Figure 18. An acceleration sensor was used to measure the cable force at the same time in the field, and its error was within 1.5%, which indicates that the tubular spot-welded grating sensor smart cable has a stable monitoring performance and can accurately monitor the working strain of the cable.

In Table 8, Fg and Fa indicate the force values for the cable obtained from the tension and test method using a fiber optic grating sensor and an accelerometer, respectively.

## 5. Conclusions

The following findings can be presented based on the design and manufacturing, mechanical performance testing, temperature testing, steel strand relaxation testing, and compensation techniques of smart steel strands and associated smart cables employing tubular spot-welded grating sensors:

(1) The tubular spot-welded grating smart cable has strong repeatability and can maintain steady and dependable adhesion. The tubular spot-welded grating sensor can detect at least 70% of the nominal breaking force of the cable.

(2) A suggested formula links the cable force to the temperature and the change in wavelength of the FBG. Under different temperatures and tensile loads, a method involving the set force, a transverse temperature coordinate, and a longitudinal strain coordinate is proposed, in which linear fitting is performed via the least squares method. The slope of the line gives a coefficient, and the calibrated force formula uses this coefficient for temperature compensation. Following temperature adjustment, the inaccuracy in the tension value is within 3.0% F.S.

(3) The relaxation rate of the epoxy-coated steel strand with the tubular spot-welded grating sensor is 4.44% when the relaxation test is carried out for 120 h, and the error between the standard load and the calculated force of the tubular spot-welded grating sensor after the relaxation test is 1.2%, indicating that the tubular spot-welded grating sensor can maintain a stable working performance under long-term loading.

(4) The test of 25 high-strength epoxy-coated steel strand wire smart cable samples yielded a maximum error in the cable force of 0.5% F.S., demonstrating the great precision and dependability of the smart cable. The discrepancy between the force of the cable measured by an acceleration sensor and the force measured on-site by the six tubular spot-welded grating sensors used for smart cables in a particular arch bridge is less than 1.5%, showing a high sensing quality.

## Figures and Tables

**Table 1:** Shear strength test results of spot-welded steel wires with epoxy coatings on stainless-steel substrates.

Test	Spot Welding Quantity/n	Tension/N	Shear Stress/MPa
1	2	54.7	35.5
2	4	118.2	38.4
3	6	174.2	37.7
Average	—	—	37.2

**Table 2:** Errors between the test and numerical values.

Standard Force/kN	9.5	30	60	90	120	150
1st wavelength/nm	1541.522	1541.983	1542.686	1543.376	1544.065	1544.752
Calculated force/kN	9.6	29.6	60.1	90.1	120	149.9
Error/%F.S.	−0.1	0.3	−0.1	−0.1	0	0.1
2nd wavelength/nm	1541.526	1541.97	1542.659	1543.342	1544.023	1544.717
Calculated force/kN	9.6	28.9	58.8	88.6	118.1	148.2
Error/%F.S.	−0.1	0.7	0.8	0.9	1.3	1.2
3rd wavelength/nm	1541.528	1541.962	1542.645	1543.324	1543.989	1544.699
Calculated force/kN	9.6	28.4	58.1	87.6	116.5	147.3
Error/%F.S.	−0.1	1.1	1.3	1.6	2.3	1.8

**Table 3:** Grating wavelength data measured under different tensile loads at different temperatures.

Load/kN	Wavelength/nm							
	−20 °C	−10 °C	0 °C	10 °C	20 °C	30 °C	40 °C	50 °C
9.5	1540.54	1540.788	1541.028	1541.276	1541.523	1541.771	1542.112	1542.412
30	1541.001	1541.249	1541.489	1541.737	1541.984	1542.232	1542.573	1542.874
60	1541.702	1541.95	1542.19	1542.438	1542.685	1542.933	1543.273	1543.574
90	1542.393	1542.641	1542.881	1543.129	1543.376	1543.624	1543.965	1544.265
120	1543.083	1543.331	1543.571	1543.819	1544.066	1544.314	1544.655	1544.956
150	1543.768	1544.016	1544.256	1544.504	1544.751	1544.999	1545.34	1545.641

**Table 4:** Comparison of the calculated force and compensated force at each temperature under each standard load.

Standard Load	9.5	30	60	90	120	107.3
−20 °C Calculated uncompensated force	−32.9	−12.8	17.5	47.6	77.6	153.3
−20 °C Calculated compensated force	13.0	33.0	63.5	93.5	123.5	118.1
−10 °C Calculated uncompensated force	−22.1	−2.1	28.4	58.4	88.4	152.6
−10 °C Calculated compensated force	12.3	32.3	62.8	92.8	122.8	128.5
0 °C Calculated uncompensated force	−11.7	8.3	38.8	68.8	98.8	151.5
0 °C Calculated compensated force	11.2	31.3	61.7	91.7	121.7	139.3
10 °C Calculated uncompensated force	−0.9	19.1	49.5	79.6	109.5	150.8
10 °C Calculated compensated force	10.5	30.6	61.0	91.0	121.0	150.0
20 °C Calculated uncompensated force	9.8	29.8	60.3	90.3	120.3	150.0
20 °C Calculated compensated force	9.8	29.8	60.3	90.3	120.3	160.8
30 °C Calculated uncompensated force	20.5	40.6	71.1	101.1	131.1	149.3
30 °C Calculated compensated force	9.1	29.1	59.6	89.6	119.6	175.7
40 °C Calculated uncompensated force	35.4	55.4	85.8	115.9	145.9	152.7
40 °C Calculated compensated force	12.4	32.4	62.8	92.9	122.9	188.7
50 °C Calculated uncompensated force	48.4	68.5	98.9	128.9	159.0	154.3
50 °C Calculated compensated force	13.9	34.0	64.4	94.5	124.5	107.3

**Table 5:** Relaxation force and relaxation rate at different times.

Test Time	Relaxation Force (kN)	Relaxation Rate/%	Test Time	Relaxation Force (kN)	Relaxation Rate/%
1 min	0.200	0.11	1 h	3.000	1.67
2 min	0.400	0.22	2 h	3.600	2.00
3 min	0.500	0.28	4 h	4.500	2.50
4 min	0.600	0.33	8 h	5.200	2.89
5 min	0.700	0.39	16 h	5.900	3.28
8 min	0.900	0.50	24 h	6.400	3.56
10 min	1.800	1.00	48 h	7.100	3.94
15 min	1.800	1.00	72 h	7.500	4.17
30 min	2.300	1.28	96 h	7.800	4.33
45 min	2.600	1.44	120 h	8.000	4.44

**Table 6:** Numerical values for the tubular spot-welded gratings during the relaxation process.

Time	Standard Load (kN)	Wavelength (nm)	Wavelength Variation (nm)	Actual Relaxation Rate
Before loading	0	1537.111	-	0.044
1 min	180.2	1541.071	3.960	
120 h	172.1	1540.884	3.773	

**Table 7:** Comparison of the cable forces in the static load test of parallel steel strand smart cables.

Standard Load (kN)	Wavelength 1# (nm)	Wavelength 2# (nm)	Average Wavelength Change (nm)	Calculated Cable Tension (kN)	Error/%F.S.
0	1562.571	1554.408	0	−10.2	−0.3
332	1562.804	1554.623	0.223	319.9	−0.4
630	1563.039	1554.821	0.44	641.0	0.4
1122	1563.385	1555.138	0.771	1130.9	0.3
1631	1563.742	1555.476	1.119	1646.0	0.5
2122	1564.075	1555.785	1.44	2121.2	0
2620	1564.428	1556.107	1.777	2620.0	0
3123	1564.773	1556.425	2.109	3111.4	−0.4

**Table 8:** Fitting equations for calibrating cable forces and field-measured cable forces.

Cable Index	Cable Force Fitting	Fg/kN	Fa/kN	Error/%
s#1	F = 2456.23Δ*λ* − 10.18	388	393	−1.3
x#1	F = 1527.70Δ*λ* + 14.30	403	409	−1.5
s#2	F = 2458.75Δ*λ* − 1.66	368	370	−0.1
x#2	F = 2927.28Δ*λ* − 17.07	379	383	−1.0
s#3	F = 1583.77Δ*λ* + 56.17	363	365	−0.1
x#3	F = 1495.78Δ*λ* − 9.57	369	371	−0.1

## Data Availability

Data is unavailable due to privacy.
